# Seven-Day Mortality Can Be Predicted in Medical Patients by Blood Pressure, Age, Respiratory Rate, Loss of Independence, and Peripheral Oxygen Saturation (the PARIS Score): A Prospective Cohort Study with External Validation

**DOI:** 10.1371/journal.pone.0122480

**Published:** 2015-04-13

**Authors:** Mikkel Brabrand, Annmarie Touborg Lassen, Torben Knudsen, Jesper Hallas

**Affiliations:** 1 Department of Medicine, Sydvestjysk Sygehus, Esbjerg, Denmark; 2 Centre South Western Denmark, Institute of Regional Health Research—University of Southern Denmark, Esbjerg, Denmark; 3 Department of Emergency Medicine, Odense University Hospital, Odense, Denmark; 4 Reseach Unit of Clinical Pharmacology, University of Southern Denmark, Odense, Denmark; Department of Transplantation and Renal Medicine, AUSTRALIA

## Abstract

**Background:**

Most existing risk stratification systems predicting mortality in emergency departments or admission units are complex in clinical use or have not been validated to a level where use is considered appropriate. We aimed to develop and validate a simple system that predicts seven-day mortality of acutely admitted medical patients using routinely collected variables obtained within the first minutes after arrival.

**Methods and Findings:**

This observational prospective cohort study used three independent cohorts at the medical admission units at a regional teaching hospital and a tertiary university hospital and included all adult (≥15 years) patients. Multivariable logistic regression analysis was used to identify the clinical variables that best predicted the endpoint. From this, we developed a simplified model that can be calculated without specialized tools or loss of predictive ability. The outcome was defined as seven-day all-cause mortality. 76 patients (2.5%) met the endpoint in the development cohort, 57 (2.0%) in the first validation cohort, and 111 (4.3%) in the second. Systolic blood Pressure, Age, Respiratory rate, loss of Independence, and peripheral oxygen Saturation were associated with the endpoint (full model). Based on this, we developed a simple score (range 0–5), ie, the PARIS score, by dichotomizing the variables. The ability to identify patients at increased risk (discriminatory power and calibration) was excellent for all three cohorts using both models. For patients with a PARIS score ≥3, sensitivity was 62.5–74.0%, specificity 85.9–91.1%, positive predictive value 11.2–17.5%, and negative predictive value 98.3–99.3%. Patients with a score ≤1 had a low mortality (≤1%); with 2, intermediate mortality (2–5%); and ≥3, high mortality (≥10%).

**Conclusions:**

Seven-day mortality can be predicted upon admission with high sensitivity and specificity and excellent negative predictive values.

## Introduction

Emergency departments and admission units across the globe are experiencing a steady increase in admissions.[1–4] Frontline personnel treating these patients must quickly assess the severity of illness. However, clinical assessment and prognostication are difficult.

Although prognostication is key to treatment selection, it is not an integrated part of modern medicine,[5] and many physicians feel inadequately trained.[6] The lack of training in prognostication adds to the importance of developing risk stratification systems that can assist in estimating the prognosis for a patient and plan treatment and resource allocation accordingly. Indeed, two studies on patients admitted to intensive care have shown that a high number of patients received inadequate care before transfer, resulting in a potential increase in mortality.[7,8]

Triage is widely used when handling high-risk patients, but the goal of triage is resource allocation,[9] not risk stratification. Several specific risk stratification systems have been introduced.[10,11] However, most of these have been developed using inadequate methodology and do not reach standards necessary for implementation in daily clinical practice.[10,11] For a system to be clinically valuable, it has to be easy to use, have adequate performance, and show reliability across groups of patients in various settings.[12]

Our objective was to develop a risk stratification system that, at admission, can accurately predict seven-day mortality of acutely admitted medical patients using routinely collected variables easily obtained within the first few minutes after arrival.

## Materials and Methods

We used multivariable logistic regression to identify the clinical variables that best predict seven-day all-cause mortality. On the basis of this, we developed a simplified model that can be calculated without special technology and without loss of performance (see Online-only Material).

We have included only parameters that are easily recorded upon admission and validated our models extensively. Only variables that provided a high prediction of outcome were included in our model, without compromising performance and reliability.

### Setting

This prospective observational cohort study consists of three independent cohorts. The development cohort was collected at the medical admission units (MAUs) at Sydvestjysk Sygehus from October 2008 through February 2009. The first validation cohort was collected from February 2010 through May 2010, and the second validation cohort at the MAU at Odense University Hospital from March 2011 through July 2011.

Sydvestjysk Sygehus Esbjerg is a regional 460-bed teaching hospital in western Denmark with a mixed urban and rural contingency population of 220 000. All subspecialties of internal medicine, pediatrics, and general and orthopedic surgery and a 12-bed intensive care unit (ICU) are present. Odense University Hospital is a 1300-bed, level 1 trauma center and a university teaching hospital with all specialties present and a contingency population of 290 000 and serves as a tertiary referral center for 1.2 million people. All adult medical patients (age 15 and older) who are admitted through the MAU (cardiology, neurology, hematology, oncology, and nephrology patients are admitted through other departments at Odense University Hospital) from all sources (ie, emergency department, family physician or out-patient clinic) were included.

### Variables

Before beginning inclusion of patients, we had selected nine potential independent variables for inclusion based upon relevancy and practical concerns: loss of independence (LOI), systolic blood pressure, age, peripheral oxygen saturation (SaO_2_), respiratory rate, level of consciousness, temperature, pulse, and blood glucose. Upon admission, a nurse registered the first collected vital signs as well as assessing LOI on a form, and the data were entered into an electronic database. During data collection, all nurses were blinded to details of the study purpose (i.e. precise endpoint and prioritized independent variables).

SaO_2_ was measured using the department’s electronic non-invasive equipment. To take the fraction of inspired oxygen (FiO_2_) into account, we used the SaO_2_/FiO_2_ ratio suggested by Rice et al.[13] and Pandharipande[14]. LOI was defined as an inability to get into bed without assistance, either from a wheelchair or emergency department/ambulance gurney, regardless of previous status. Level of consciousness was recorded using the AVPU (defined as Alert, responsive to Vocal stimuli, responsive to Pain, or Unresponsive) scale.[15,16]

### Endpoint

The endpoint was all-cause seven-day mortality regardless of admission status, co-morbidity, and “do not attempt resuscitation” orders. Data on the endpoint were extracted from the Danish Person Register[17] and retrieved after all patients were discharged. Foreign nationals (n = 50; 0.6%) who were discharged alive were considered to be alive at the endpoint, even though complete follow-up was impossible.

### Ethics

The study was approved by the Danish Data Protection Agency and reported in accordance with the STROBE statement.[18] Danish law does not require approval by the regional ethics committee for observational studies.

### Statistics

To reduce the risk of overfitting,[19–21] we required 10 events per independent variable, ie, 90, to include all predefined variables. In case of fewer events, we needed to reduce the number of independent variables. Before beginning analyses, we decided that LOI, systolic blood pressure, age, and respiratory rate would remain, based on the existing literature. We determined that blood glucose could be discarded (because it is easily lowered and increased), as could temperature because it can be measured in various ways (eg, tympanic, axillary, and rectal), which could affect predictions.[22] If further variables were to be discarded, we prioritized level of consciousness, peripheral oxygen saturation, and lowest, pulse.

Both the full and the simple models were developed using only patients from the development cohort. Both models were afterwards validated independently in the validation cohorts using coefficients and scores as identified in the development cohort (see S1 Text).

### Generation of the full model

We analyzed the association between the independent variables and the endpoint using univariable analyses with a 25% significance level. The variables were included in a multivariable logistic regression analysis with a 5% significance level. We tested for interaction, co-linearity and deviation from linearity using fractional polynomials in the continuous variables.[23] To minimize the impact of missing values, we used multiple imputation (data considered to be missing at random)[24–26] in our main analyses and report these coefficients.

### Generation of the simplified model

To develop a model that would be easy to use in clinical practice and make mental calculation possible, we defined a simplified model by dichotomizing the continuous variables included in the full model. The cutoff level for dichotomization was arbitrarily defined as the point at which the mortality of each variable rose above 5%. Because SaO_2_/FiO_2_ is difficult to calculate mentally, we defined the threshold as SaO_2_ below the 5% mortality level on room air or if the patient received any supplementary oxygen.

### Performance of the models

Discriminatory power (the ability to identify the participants at highest risk) for both the full and simplified models was assessed using area under the receiver-operating characteristic curve (AUROC).[27] Calibration (ie, the ability to correctly estimate risk of death) was tested using the Hosmer-Lemeshow goodness-of-fit test[28] for the full model and Pearson’s χ^2^ goodness-of-fit test for the simplified model. To further explore the calibration of our simplified model, we decided to replicate the method introduced by Seymour et al.[29] Briefly, we first predicted the probabilities of the individual scores using logistic regression analysis and then calculated the Hosmer-Lemeshow goodness-of-fit test.

Discriminatory power was considered to be excellent when AUROC was over 0.8,[28] and calibration was considered acceptable when the goodness-of-fit test reached *P*>05.[28]

### Sensitivity analysis

We planned an extensive set of sensitivity analyses. Our primary concern was missing data, and we reran the analysis using list-wise deletion and imputation of the mean instead of multiple imputation.[24–26]

Development of our full model was not automated and could potentially be affected by irrational preferences. We performed an automated model development using stepwise regression with backward elimination initially using both all nine potential independent variables and only the prioritized variables (in case of too few events).

LOI is not widely used in risk stratification, and there is no generally accepted definition. We thus tested two other markers, ie, inability to stand unaided[30] and inability to rise from a chair unaided.[31]

Use of SaO_2_/FiO_2_ is new in this context. For this reason, we introduced the partial pressure of O_2_ (PaO_2_)/FiO_2_ as an alternative, as suggested by Rice et al. and Pandharipande.[13,14] PaO_2_ was estimated using linear regression.

Our arbitrary choice of a 5% cutoff for the dichotomization in the simplified model was not based on statistical calculation. As an alternative, we applied a 10% cutoff.

Last, we recalculated the simplified model under the assumption that missing values of the variables in the score were normal, ie, that they had a score of 0.

### Sample size and descriptive statistics

To define the sample size, we required 90 cases if we were to include nine independent variables.[19–21] With an estimated 3% mortality, we required 3000 cases in the development cohort.

Data are reported as mean (standard deviation [SD]) or proportions whenever appropriate, with the 95% confidence interval (CI) when applicable. Stata version 12.1 (Stata Corp LP, College Station, Texas, USA) was used for analyses.

## Results

We had 3046 admissions (2608 patients) in the development cohort; 2848 (2463 patients) in the first validation cohort; 2561 (2210 patients) in the second validation cohort; and all were included in the study. Seventy-six patients (2.5%) died within seven days from admission in the development cohort, as did 57 patients (2.0%) in the first validation cohort and 111 (4.3%) in the second. Patients who died had a higher age, pulse, blood glucose, and respiratory rate but a lower systolic blood pressure, temperature, and SaO_2_/FiO_2_ while fewer were alert and more had lost their independence. Characteristics of the admissions can be found in Table 1.

**Table 1:** Demographic information on participants, mean (SD) unless otherwise stated;—indicates data not available or relevant.

Variable	Development cohort			First validation cohort			Second validation cohort		
	Total (n = 3046)	Alive (n = 2970)	Dead (n = 76)	Total (n = 2848)	Alive (n = 2791)	Dead (n = 57)	Total (n = 2561)	Alive (n = 2450)	Dead (n = 111)
Age (years)	62.4 (19.2)	62.1 (19.2)	74.3 (15.2)	61.1 (19.4)	60.8 (19.4)	77.5 (10.6)	63.0 (20.8)	62.2 (20.9)	78.9 (10.9)
Female (%)	1460 (47.9)	1426 (48.0)	34 (44.7)	1490 (52.3)	1460 (52.3)	30 (52.6)	1379 (53.9)	1321 (53.9)	58 (52.3)
Pulse (beats/min)	88.2 (22.2)	88.0 (22.0)	94.8 (25.4)	86.4 (20.6)	86.2 (20.5)	94.5 (25.4)	87.9 (19.8)	87.6 (19.4)	97.0 (26.1)
Systolic blood pressure (mmHg)	134.0 (24.8)	134.5 (24.6)	116.3 (26.1)	139.2 (26.5)	139.5 (26.3)	125.5 (29.3)	132.6 (26.2)	133.2 (25.9)	119.4 (28.2)
Temperature (°C)	37.0 (0.9)	37.1 (0.8)	36.8 (1.2)	36.9 (0.8)	36.9 (0.8)	36.7 (1.1)	37.4 (0.9)	37.4 (0.9)	37.2 (1.2)
SaO_2_/FiO_2_ (%/100)	424.7 (68.8)	426.7 (66.0)	344.6 (116.1)	433.1 (69.4)	434.4 (67.9)	368.2 (107.1)	431.0 (66.4)	434.9 (60.7)	337.7 (113.6)
Respiratory rate (breaths/min)	19.3 (6.2)	19.1 (6.1)	25.5 (8.6)	17.5 (5.8)	17.4 (5.7)	23.3 (9.3)	19.4 (6.2)	19.1 (5.9)	26.3 (9.2)
Blood glucose (mmol/L)	7.1 (3.6)	7.1 (3.5)	7.9 (4.3)	7.2 (3.4)	7.2 (3.4)	9.1 (5.3)	-	-	-
Level of consciousness (AVPU), n (%)	Alert: 2853 (94.2) Vocal: 124 (4.1) Pain: 37 (1.2) Unresponsive: 14 (0.5)	Alert: 2796 (94.6) Vocal: 115 (3.9) Pain: 34 (1.2) Unresponsive: 10 (0.3)	Alert: 57 (78.1) Vocal: 9 (12.3) Pain: 3 (4.1) Unresponsive: 4 (5.5)	Alert: 2668 (95.1) Vocal: 103 (3.7) Pain: 14 (0.5) Unresponsive: 22 (0.8)	Alert: 2627 (95.5) Vocal: 92 (3.3) Pain: 13 (0.5) Unresponsive: 19 (0.7)	Alert: 41 (73.2) Vocal: 11 (19.6) Pain: 1 (1.8) Unresponsive: 3 (5.4)	Alert: 2266 (90.6) Vocal: 161 (6.4) Pain: 43 (1.7) Unresponsive: 30 (1.2)	Alert: 2195 (91.7) Vocal: 143 (6.0) Pain: 37 (1.6) Unresponsive: 19 (0.8)	Alert: 71 (67.0) Vocal: 18 (17.0) Pain: 6 (5.7) Unresponsive: 11 (10.4)
Loss of independence (yes/no), n (%)	567 (22.8)	515 (21.2)	52 (82.5)	576 (22.4)	530 (21.0)	46 (88.5)	950 (38.9)	854 (36.6)	96 (90.6)
7-day mortality (%), n (%)	76 (2.5)	-	-	57 (2.0)	-	-	111 (4.3)	-	-
7-day in-hospital mortality (%), n (%)	65 (56.0)	-	-	51 (57.3)	-	-	100 (67.6)	-	-
Length of stay (days)	4.7 (9.8)	4.7 (9.9)	2.6 (2.3)	4.5 (9.1)	4.6 (9.2)	2.6 (2.2)	5.2 (7.5)	5.3 (7.7)	3.1 (2.3)
Missing data on any of the nine independent variables, n (%)	1220 (40.1)	1186 (39.9)	34 (44.8)	726 (25.5)	708 (25.4)	18 (31.6)	-	-	-
Missing data on any of the five included independent variables, n (%)	1062 (34.9)	1036 (34.9)	26 (34.2)	587 (20.6)	570 (20.4)	17 (29.8)	595 (23.2)	563 (23.0)	32 (28.9)

### Development of the full model

We could, according to the number of outcomes (fewest in the first validation cohort), analyze six independent variables and had, as previously stated, prioritized LOI, systolic blood pressure, age, SaO_2_/FiO_2_, respiratory rate, and level of consciousness. All were associated with the endpoint in univariable analyses.

Using multivariable logistic regression, we found systolic blood pressure, age, respiratory rate, SaO_2_/FiO_2_, and LOI to be associated with the endpoint whereas loss of consciousness was not (see S1 Table). We did not identify interaction between variables and found no evidence of deviation from linearity (see also S1 Text). The full model is presented in Table 2.

**Table 2:** Results of model development for both the full and simplified models (PARIS score). For the full model, we provide both exact coefficients and odds ratios.

Variable	Full model, coefficients	Full model, odds ratios	PARIS score, cutoffs
Systolic blood pressure (mmHg)-	-0.025 (-0.036–0.014)		≤115
Per 10 mmHg		0.78 (0.70–0.87)	
Age (years)	0.024 (0.0059–0.043)		≥80
- Per 10 years		1.28 (1.08–1.53)	
Respiratory rate (breaths/min)	0.042 (0.0076–0.077)		≥25
- Per 5 breaths		1.23 (1.04–1.47)	
SaO_2_/FiO_2_ (%/100)	-0.0044 (-0.0071–0.0017)		≤93% (SaO_2_) or any supplemental oxygen
- Per 50 (%/100)		0.80 (0.70–0.92)	
Loss of independence (yes/no)	1.6 (0.90–2.3)	4.96 (2.45–10.06)	Yes
Intercept	-2.2 (-4.6–0.091)	-	-

### Development of the simplified model

Mortality rose above 5% when systolic blood pressure was ≤115 mmHg, age ≥80 years, respiratory rate ≥25 breaths per minute, and SaO_2_ ≤93%. These limits, any use of supplementary oxygen, and LOI were used as cutoffs in our simplified model, allowing for a score ranging from 0–5 (Table 2). We named our simplified model the PARIS score, derived from systolic blood Pressure, Age, Respiratory rate, loss of Independence and peripheral oxygen Saturation.

### Sensitivity analyses

Our sensitivity analyses did not lead to improvement or major deviations from our models (see S2, S3, S4 and S5 Tables).

### Performance of the models

The discriminatory power was excellent (AUROC≥0.87) and the calibration good for the full model in all cohorts (Table 3). In the PARIS score, we found excellent discriminatory power (AUROC≥0.86) in all cohorts, and calibration was acceptable in the first validation cohort but failed in the second validation cohort (Table 3).

**Fig 1:**
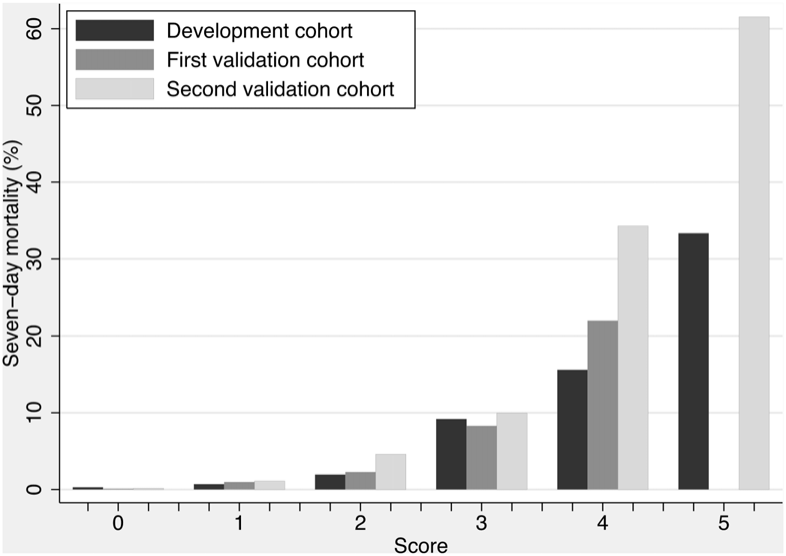
Score and seven-day mortality in the simplified model (PARIS score) in all three cohorts, *P* for trend within cohorts <0.001. Approximately 1000 patients had a score of 0; 800 a score of 1; 500 a score of 2; 250 a score of 3; 70 a score of 4; and 10 a score of 5 in the three cohorts.

In the PARIS score, seven-day mortality increased with increasing score (Fig 1). With a score of three or higher, sensitivity was 74.0%, specificity 85.9%, positive predictive value 11.9%, and negative predictive value 99.2% in the development cohort. Sensitivity was lower in the validation cohorts, specificity was slightly higher, and the negative predictive value remained high (Table 4). Patients with score ≤1 had mortality ≤1.1%; with 2, mortality was 1.9–4.6%; and ≥3, mortality was ≥8.3% (S6 and S7 Tables).

**Table 3:** Performance measures of the models, both discriminatory power (ability to identify patients at increased risk) and calibration (precision in predictions).

Measure	Full model	Simplified model (PARIS score)
Discriminatory power		
- Development cohort	0.87 (0.82–0.93)	0.86 (0.80–0.91)
- First validation cohort	0.90 (0.87–0.93)	0.87 (0.82–0.92)
- Second validation cohort	0.88 (0.84–0.91)	0.86 (0.82–0.90)
Calibration—Hosmer-Lemeshow goodness of fit		
- Development cohort	*P* = 0.97	*P* = 0.42
- First validation cohort	*P* = 0.75	*P* = 0.74
- Second validation cohort	*P* = 0.33	*P*<0.001
Pearson’s χ^2^ goodness of fit		
- Development cohort	*-*	*-*
- First validation cohort	*-*	*P* = 0.42
- Second validation cohort	*-*	*P*<0.001

**Table 4:** Classification function of the simplified model (PARIS score). Data are specified for all three cohorts at a score ≥3, identified as the optimal cutoff.

	Score ≥1	Score ≥2	Score ≥3			Score ≥4	Score = 5
	Development cohort	Development cohort	Development cohort	First validation cohort	Second validation cohort	Development cohort	Development cohort
True positives	48	44	37	25	55	17	5
False negatives	2	6	13	15	29	33	45
True negatives	724	1305	1661	2023	1703	1859	1924
False positives	1210	629	273	198	259	75	10
Sensitivity	96 (86.3–99.5)	88 (75.7–95.5)	74 (59.7–85.4)	62.5 (45.8–77.3)	65.5 (54.3–75.5)	34 (21.2–48.8)	10 (3.33–21.8)
Specificity	37.4 (35.3–39.6)	67.5 (65.3–69.6)	85.9 (84.3–87.4)	91.1 (89.8–92.2)	86.8 (85.2–88.3)	96.1 (95.2–96.9)	99.5 (99.1–99.8)
Positive predictive value	3.82 (2.83–5.03)	6.54 (4.79–8.68)	11.9 (8.54–16.1)	11.2 (7.4–16.1)	17.5 (13.5–22.2)	18.5 (11.1–27.9)	33.3 (11.8–61.6)
Negative predictive value	99.7 (99.0–100.0)	99.5 (99.0–99.8)	99.2 (98.7–99.6)	99.3 (98.8–99.6)	98.3 (97.6–98.9)	98.3 (97.6–98.8)	97.7 (97.0–98.3)

### Discussion

We have developed and validated a risk stratification system that can predict seven-day all-cause mortality for acutely admitted medical patients. Using five easily obtainable variables (ie, systolic blood pressure, age, respiratory rate, peripheral oxygen saturation [corrected for the fraction of inspired oxygen], and LOI), we have shown that an important outcome can be predicted at the time of admission with high accuracy.

Use of risk stratification tools might help the clinician but is not without important limitations. Statistics, chance, and human perseverance dictate that even the best risk stratification system will not be completely accurate and patients predicted to be at low risk might eventually die. This is one reason why authors have advocated that these systems should be used with caution on individual patients,[32–35] as our data remind us. Even with a cutoff of 1, two patients in the development cohort would have been designated as low risk yet still died (Table 4).

Clinical assessment relying on experience alone is an interesting alternative to complex models. However, clinical assessment alone has never been scientifically proven as a strong predictive tool in an admission unit. Data from other environments suggest that it has limitations. Comparing a clinician gut feeling to clinical features (eg, medical history, observation, and clinical examination), Van den Bruel et al. found that gut feeling could identify sick children missed by clinical features at a cost of decreased specificity.[36] Asking attending physicians, residents, and nurses to predict in-hospital mortality of medical ICU patients, Meadow et al. found a high level of discordant predictions, and only 52% of the patients predicted to die actually died while 15% survived unexpectedly.[37] Our PARIS score is not perfect either. Use without critical evaluation will lead to cases being missed. If the suggested cutoff of ≥3 is implemented, 13–29 patients will be missed and 198–273 falsely identified. Development of more accurate models is needed.

Compared to clinical experience, risk stratification systems have some advantages. First of all, they are expected to have better intra- and inter-observer reliability because fewer parameters are subject to interpretation. Second, they should have improved external validity because they do not require exactly the same clinicians to be present at each institution to make the prediction. Last, most scores can be calculated automatically once the staff has collected the information. The predicted mortality could then be added to the overall picture and provide another piece of the puzzle for the physician. At this point, we do not know to which degree risk stratification systems supplement performance in clinical practice, and further studies are warranted.

We provide two models, a complex (full) model with a precise prediction of mortality and a simplified model with a score for seven-day mortality (the PARIS score). Both models have their place in a MAU. The full model, although precise, is difficult to calculate and requires computational support. Discriminatory power is excellent and calibration good even in an external environment. We believe that the full model is best suited for research purposes (eg, comparing cohorts). The PARIS score can easily be calculated mentally. Discriminatory power is excellent, but calibration in an external environment was not perfect. However, increasing mortality follows increasing scores (Fig 1), and we believe that the PARIS score can be used as an additional tool in identifying patients at increased risk of poor outcome.

The external validity of our models is good. We included all patients admitted, not only patients thought to be of either high or low risk or other select characteristics. Our models have been through rigorous statistical analyses and, most important, validated externally. Our second validation cohort is a completely independent sample from an institution far removed from our own, not only geographically but also in time and in terms of case-mix. In both validation cohorts, the nursing staffs were given a short written and oral introduction to the variables assessed and were fully able to register the necessary information. To further test the generalizability of our score, dr. John Kellett of Nenagh Hospital in Ireland has kindly validated our simplified score. He found a discriminatory power of 0.803 and acceptable calibration (p = 0.08) in an Irish sample and a discriminatory power of 0.714 and good calibration (p = 0.27) in a Ugandan cohort from Kitovu Hospital (personal communication).

The difference in case-mix (ie, mortality) between the two institutions would serve to explain the differences in negative and positive predictive values (as well as calibration) in the second validation cohort. With mortality almost twice as high (for multifactorial reasons, eg, access to outpatient evaluation, proportion of urban population, and decision to admit made by attending rather than resident physicians), this scenario is expected.

Our study has limitations and weaknesses. First, we were affected by missing data (especially LOI and respiratory rate), and to compensate, we used multiple imputation. However, our extensive sensitivity analyses proved that this was not a problem. Second, we had a limited case-mix because we have evaluated our models only on medical patients. However, within this spectrum, our models have proven to be reliable although they still must be tested on surgical patients. Also, our first two cohorts are very similar. Only the second validation cohort differs significantly. Therefore, further validation in lager groups of medical patients is warranted. Third, use of LOI is unconventional. It is not routinely documented, but we decided to include it regardless because previous studies have shown that its inclusion improves models.[10] Fourth, our model is limited by not including specific variables on co-morbidity and physical capacity. To compensate, we added LOI as this can be seen as a general marker of capability. Last, we have not assessed inter-observer reliability of our models or tested reproducibility.

From a patient, clinician, and organizational perspective, a risk stratification model has no meaning in itself. The true value lies in its ability to guide the clinician to deliver improved care. The optimal measure would be reduced seven-day mortality after implementation, but we have not performed an impact analysis; therefore, we still need to test whether our model will improve patient care.

## Conclusions

We have shown and validated that seven-day all-cause mortality can be predicted with excellent discriminatory power and acceptable calibration upon admission for acutely admitted medical patients. Before our models should be used in clinical practice, there still is a need for further independent validation studies as well as a randomized trial to evaluate patient outcome when the scoring system is used.

## Supporting Information

S1 Text Additional methods. (DOCX)

S1 Table Internal validation in the development cohort using bootstrapping with 1984 replications. (DOCX)

S2 Table Logistic regression using two alternative definitions of loss of independence, ie, ability to stand unaided and unable to get out of a chair unaided. (DOCX)

S3 Table Performance measures using two alternative definitions of loss of independence, ie, ability to stand unaided and unable to get out of a chair unaided. (DOCX)

S4 Table Missing data in all three cohorts, data presented as number (%). (DOCX)

S5 Table Logistic regression of the full model using list-wise deletion without multiple imputation. (DOCX)

S6 Table Seven-day mortality in the simplified model in each of the three cohorts, number (%). (DOCX)

S7 Table Logistic regressions of the simplified score, both univariable and multivariable analyses; CI, confidence interval. (DOCX)

## References

[1] Statistics Denmark. Sygehus Benyttelse (2013) Available: http://www.dst.dk/da/Statistik/emner/sundhed/sygehusbenyttelse.aspx

[2] Lowthian JA, Curtis AJ, Cameron PA, Stoelwinder JU, Cooke MW, McNeil JJ (2011) Systematic review of trends in emergency department attendances: an Australian perspective. Emerg Med J 28: 373–377. 10.1136/emj.2010.099226 20961936

[3] Pitts SR, Pines JM, Handrigan MT, Kellermann AL (2012) National trends in emergency department occupancy, 2001 to 2008: effect of inpatient admissions versus emergency department practice intensity. Ann Emerg Med 60: 679–686 e673 10.1016/j.annemergmed.2012.05.014 22727201

[4] Wai AK, Chor CM, Lee AT, Sittambunka Y, Graham CA, Rainer TH (2009) Analysis of trends in emergency department attendances, hospital admissions and medical staffing in a Hong Kong university hospital: 5-year study. Int J Emerg Med 2: 141–148. 10.1007/s12245-009-0098-7 20157463 PMC2760706

[5] Christakis NA (1997) The ellipsis of prognosis in modern medical thought. Soc Sci Med 44: 301–315. 9004366 10.1016/s0277-9536(96)00100-1

[6] Christakis NA, Iwashyna TJ (1998) Attitude and self-reported practice regarding prognostication in a national sample of internists. Arch Intern Med 158: 2389–2395. 9827791 10.1001/archinte.158.21.2389

[7] McGloin H, Adam SK, Singer M (1999) Unexpected deaths and referrals to intensive care of patients on general wards. Are some cases potentially avoidable? J R Coll Physicians Lond 33: 255–259. 10402575 PMC9665643

[8] McQuillan P, Pilkington S, Allan A, Taylor B, Short A, Morgan G, et al (1998) Confidential inquiry into quality of care before admission to intensive care. BMJ 316: 1853–1858. 9632403 10.1136/bmj.316.7148.1853 PMC28582

[9] Wuerz RC, Milne LW, Eitel DR, Travers D, Gilboy N (2000) Reliability and validity of a new five-level triage instrument. Acad Emerg Med 7: 236–242. 10730830 10.1111/j.1553-2712.2000.tb01066.x

[10] Brabrand M, Folkestad L, Clausen NG, Knudsen T, Hallas J (2010) Risk scoring systems for adults admitted to the emergency department: a systematic review. Scand J Trauma Resusc Emerg Med 18: 8 10.1186/1757-7241-18-8 20146829 PMC2835641

[11] Siontis GC, Tzoulaki I, Ioannidis JP (2011) Predicting death: an empirical evaluation of predictive tools for mortality. Arch Intern Med 171: 1721–1726. 10.1001/archinternmed.2011.334 21788535

[12] McGinn TG, Guyatt GH, Wyer PC, Naylor CD, Stiell IG, Richardson WS (2000) Users' guides to the medical literature: XXII: how to use articles about clinical decision rules. Evidence-Based Medicine Working Group. JAMA 284: 79–84. 10872017 10.1001/jama.284.1.79

[13] Rice TW, Wheeler AP, Bernard GR, Hayden DL, Schoenfeld DA, Ware LB (2007) Comparison of the SpO2/FIO2 ratio and the PaO2/FIO2 ratio in patients with acute lung injury or ARDS. Chest 132: 410–417. 17573487 10.1378/chest.07-0617

[14] Pandharipande PP, Shintani AK, Hagerman HE, St Jacques PJ, Rice TW, Sanders NW, et al (2009) Derivation and validation of Spo2/Fio2 ratio to impute for Pao2/Fio2 ratio in the respiratory component of the Sequential Organ Failure Assessment score. Crit Care Med 37: 1317–1321. 10.1097/CCM.0b013e31819cefa9 19242333 PMC3776410

[15] Kelly CA, Upex A, Bateman DN (2004) Comparison of consciousness level assessment in the poisoned patient using the alert/verbal/painful/unresponsive scale and the Glasgow Coma Scale. Ann Emerg Med 44: 108–113. 15278081 10.1016/j.annemergmed.2004.03.028

[16] McNarry AF, Goldhill DR (2004) Simple bedside assessment of level of consciousness: comparison of two simple assessment scales with the Glasgow Coma scale. Anaesthesia 59: 34–37. 14687096 10.1111/j.1365-2044.2004.03526.x

[17] Pedersen CB (2011) The Danish Civil Registration System. Scand J Public Health 39: 22–25. 10.1177/1403494810387965 21775345

[18] Vandenbroucke JP, von Elm E, Altman DG, Gotzsche PC, Mulrow CD, Pocock SJ, et al (2007) Strengthening the Reporting of Observational Studies in Epidemiology (STROBE): explanation and elaboration. Epidemiology 18: 805–835. 18049195 10.1097/EDE.0b013e3181577511

[19] Peduzzi P, Concato J, Feinstein AR, Holford TR (1995) Importance of events per independent variable in proportional hazards regression analysis. II. Accuracy and precision of regression estimates. J Clin Epidemiol 48: 1503–1510. 8543964 10.1016/0895-4356(95)00048-8

[20] Peduzzi P, Concato J, Kemper E, Holford TR, Feinstein AR (1996) A simulation study of the number of events per variable in logistic regression analysis. J Clin Epidemiol 49: 1373–1379. 8970487 10.1016/s0895-4356(96)00236-3

[21] Concato J, Feinstein AR, Holford TR (1993) The risk of determining risk with multivariable models. Ann Intern Med 118: 201–210. 8417638 10.7326/0003-4819-118-3-199302010-00009

[22] Sener S, Karcioglu O, Eken C, Yaylaci S, Ozsarac M (2012) Agreement between axillary, tympanic, and mid-forehead body temperature measurements in adult emergency department patients. Eur J Emerg Med 19: 252–256. 10.1097/MEJ.0b013e32834c5841 21945968

[23] Sauerbrei W, Royston P (1999) Building multivariable prognostic and diagnostic models: transformation of the predictors by using fractional polynomials. J R Statist Soc 162: 71–94.

[24] Marshall A, Altman DG, Royston P, Holder RL (2010) Comparison of techniques for handling missing covariate data within prognostic modelling studies: a simulation study. BMC Med Res Methodol 10: 7 10.1186/1471-2288-10-7 20085642 PMC2824146

[25] Schafer JL, Graham JW (2002) Missing data: our view of the state of the art. Psychol Methods 7: 147–177. 12090408

[26] Sterne JA, White IR, Carlin JB, Spratt M, Royston P, Kenward MG, et al (2009) Multiple imputation for missing data in epidemiological and clinical research: potential and pitfalls. BMJ 338: b2393 10.1136/bmj.b2393 19564179 PMC2714692

[27] Hanley JA, McNeil BJ (1982) The meaning and use of the area under a receiver operating characteristic (ROC) curve. Radiology 143: 29–36. 7063747 10.1148/radiology.143.1.7063747

[28] Hosmer DW, Lemeshow S (2000) Applied logistic regression. New York, USA: John Wiley & Sons.

[29] Seymour CW, Kahn JM, Cooke CR, Watkins TR, Heckbert SR, Rea TD (2010) Prediction of critical illness during out-of-hospital emergency care. JAMA 304: 747–754. 10.1001/jama.2010.1140 20716737 PMC3949007

[30] Kellett J, Deane B (2006) The Simple Clinical Score predicts mortality for 30 days after admission to an acute medical unit. QJM 99: 771–781. 17046859 10.1093/qjmed/hcl112

[31] Kellett J, Deane B, Gleeson M (2008) Derivation and validation of a score based on Hypotension, Oxygen saturation, low Temperature, ECG changes and Loss of independence (HOTEL) that predicts early mortality between 15 min and 24 h after admission to an acute medical unit. Resuscitation 78: 52–58. 10.1016/j.resuscitation.2008.02.011 18406038

[32] Lemeshow S, Klar J, Teres D (1995) Outcome prediction for individual intensive care patients: useful, misused, or abused? Intensive Care Med 21: 770–776. 8847434 10.1007/BF01704747

[33] Teres D, Lemeshow S (1994) Why severity models should be used with caution. Crit Care Clin 10: 93–110; discussion 111–115. 8118735

[34] Zollo MB, Moskop JC, Kahn CE Jr. (1996) Knowing the score: using predictive scoring systems in clinical practice. Am J Crit Care 5: 147–151. 8653166

[35] (1997) Consensus statement of the Society of Critical Care Medicine's Ethics Committee regarding futile and other possibly inadvisable treatments. Crit Care Med 25: 887–891. 9187612 10.1097/00003246-199705000-00028

[36] Van den Bruel A, Thompson M, Buntinx F, Mant D (2012) Clinicians' gut feeling about serious infections in children: observational study. BMJ 345: e6144 10.1136/bmj.e6144 23015034 PMC3458229

[37] Meadow W, Pohlman A, Frain L, Ren Y, Kress JP, Teuteberg W, et al (2011) Power and limitations of daily prognostications of death in the medical intensive care unit. Crit Care Med 39: 474–479. 10.1097/CCM.0b013e318205df9b 21150582

