# Supplementary material for: Seven-Day Mortality Can Be Predicted in Medical Patients by Blood Pressure, Age, Respiratory Rate, Loss of Independence, and Peripheral Oxygen Saturation (the PARIS Score): A Prospective Cohort Study with External Validation
Source: PLoS One. 2015 Apr 13;10(4):e0122480. doi: 10.1371/journal.pone.0122480 (PMC4395094; doi:10.1371/journal.pone.0122480)
Supplement: S1 Text — (DOCX) [file pone.0122480.s001.docx]

**S1 Text – Additional methods**

**Development of the full model**

AVPU

Loss of consciousness was originally included as an AVPU (ie, Alert, responsive to Vocal stimuli, responsive to Pain, and Unresponsive) score. This was not found to be significantly associated with the endpoint in multivariable analysis. We therefore redefined AVPU to the dichotomized variable of alert or not. We also tested a dichotomization of the combination of alert and responsive to vocal stimulation or not. Neither of these two was significantly associated with the endpoint in multivariable analysis, and inclusion in the model did not result in improvement.

Interaction

We found no indication of interaction among the variables. However, an interaction between age and systolic blood pressure was borderline significant with *P*=.0505 using the likelihood-ratio test. Nevertheless, adding this interaction term to the model did not result in improvement.

Deviation from linearity

Our primary analysis of deviation from linearity was using fractional polynomials.[1] As mentioned, we found no evidence of this. As part of our sensitivity analysis, we tested if using restricted cubic splines or linear splines could model our endpoint better. Using the likelihood-ratio test, we found a significant effect of modeling systolic blood pressure using restricted cubic splines. However, when testing using the Akaike information criterion,[2] discrimination, and calibration, we found no significant effect, and systolic blood pressure was kept in the linear term.

Co-linearity

We found no evidence of co-linearity between the included variables.

Internal validation

As a test of internal validation, we performed bootstrap logistic regression with 1984 replications. All variables included in the full model retained their significant association with the endpoint; see eTable 1.

Sensitivity analyses

Of the five independent variables of the full model, all were significantly associated with seven-day mortality in the validation cohorts in multivariable logistic regression analyses, except systolic blood pressure (*P*=.137) and SaO_2_/FiO_2_ (*P*=.062) in the first validation cohort.

We collected data in the development and first validation cohorts on two other measurements of loss of independence, ie, the ability to stand unaided and the ability to get out of a chair without assistance. Using these instead of the ability to get into bed did not improve the model; see eTables 2 and 3.

As an alternative to SaO_2_/FiO_2_, we performed a linear regression to predict the partial pressure of O_2_ (PaO_2_) corrected for FiO_2_ using SaO_2_. This did not improve our model.

Our full model was developed using a manual technique. To reduce potential interference by irrational preferences, we also performed an automated model development using stepwise logistic regression with backwards elimination. When we included all nine potential independent variables, systolic blood pressure, age, respiratory rate, SaO_2_/FiO_2_, loss of independence, and temperature were all associated with the endpoint. However, discriminatory power was unaffected, and calibration failed in the second validation cohort. Using stepwise logistic regression with backward elimination, including only the six preselected variables due to the number of events, resulted in a model identical to our manually populated version.

The choice to use multiple imputation was based on the amount of missing data; see eTable 4. If we instead used list-wise deletion without multiple imputation, we arrived at the same model but with a slightly wider confidence interval in the coefficients; see eTable 5.

**Development of the simplified model**

In all three cohorts, we observed a steady increase in mortality with increasing scores; see eTable 6.

Calibration of the simplified model

As stated, we wished to expand our evaluation of the calibration of the simplified model. We therefore, according to Seymour et al.,[3] performed a univariable logistic regression of the simplified model (see eTable 7) and used this to calculate the Hosmer-Lemeshow goodness-of-fit test. We also performed a multivariable logistic regression of the simplified model; see eTable 7.

Sensitivity analysis of the simplified model

To ensure the validity of the simplified score, we calculated an alternative version by rounding the coefficients of a categorized version of each of the continuous variables to the nearest integer. This resulted in a model inferior to the presented score.

Because the overall seven-day mortality in the second validation cohort was approximately 5%, we calculated a simplified model using 10% mortality as the cutoff. This did not improve the calibration.

We also used the assumption that an unknown value of part of the score was normal. This did not improve the model.

**References**

1. Royston P, Ambler G, Sauerbrei W (1999) The use of fractional polynomials to model continuous risk variables in epidemiology. Int J Epidemiol 28: 964-974.

2. Bozdogan Z (1987) Model selection and Akaike's information criterion (AIC): The general theory and its analytical extesions. Psychometrika 52: 345-370.

3. Seymour CW, Kahn JM, Cooke CR, Watkins TR, Heckbert SR, Rea TD (2010) Prediction of critical illness during out-of-hospital emergency care. JAMA 304: 747-754.
