# Supplementary material for: Seven-Day Mortality Can Be Predicted in Medical Patients by Blood Pressure, Age, Respiratory Rate, Loss of Independence, and Peripheral Oxygen Saturation (the PARIS Score): A Prospective Cohort Study with External Validation
Source: PLoS One. 2015 Apr 13;10(4):e0122480. doi: 10.1371/journal.pone.0122480 (PMC4395094; doi:10.1371/journal.pone.0122480)
Supplement: S1 Table — (DOCX) [file pone.0122480.s002.docx]

**S1 Table - Internal validation in the development cohort using bootstrapping with 1984 replications**

| Variable | Coefficient | 95% confidence interval | *P* value |
| --- | --- | --- | --- |
| Systolic blood pressure | -0.025 | -0.040-0.0099 | .001 |
| Age | 0.025 | 0.0014-0.049 | .038 |
| Respiratory rate | 0.049 | 0.011-0.087 | .011 |
| SaO_2_/FiO_2_ | -0.0044 | -0.0076-0.0011 | .008 |
| Loss of independence | 1.70 | 0.93-2.47 | <.001 |
| Intercept | -2.44 | -5.70-0.82 | .143 |
