# Supplementary material for: Seven-Day Mortality Can Be Predicted in Medical Patients by Blood Pressure, Age, Respiratory Rate, Loss of Independence, and Peripheral Oxygen Saturation (the PARIS Score): A Prospective Cohort Study with External Validation
Source: PLoS One. 2015 Apr 13;10(4):e0122480. doi: 10.1371/journal.pone.0122480 (PMC4395094; doi:10.1371/journal.pone.0122480)
Supplement: S2 Table — (DOCX) [file pone.0122480.s003.docx]

**S2 Table - Logistic regression using two alternative definitions of loss of independence, ie, ability to stand unaided and unable to get out of a chair unaided**

| Unable to stand unaided | | | |
| --- | --- | --- | --- |
| Variable | Coefficient | 95% confidence interval | *P* value |
| Systolic blood pressure | -0.024 | -0.037-0.010 | <.001 |
| Age | 0.026 | 0.0048-0.048 | .017 |
| Respiratory rate | 0.057 | 0.017-0.096 | .005 |
| Loss of independence | 1.64 | 0.90-2.37 | <.001 |
| SaO_2_/FiO_2_ | -0.0045 | -0.0076-0.0013 | .006 |
| Intercept | -2.81 | -5.58-0.03 | .048 |
| Unable to get out of chair unaided | | | |
| Systolic blood pressure | -0.023 | -0.037-0.010 | .001 |
| Age | 0.024 | 0.0029-0.046 | .026 |
| Respiratory rate | 0.054 | 0.015-0.094 | .007 |
| Loss of independence | 1.84 | 1.07-2.60 | <.001 |
| SaO_2_/FiO_2_ | -0.0040 | -0.0072-0.00070 | .015 |
| Intercept | -2.92 | -5.71-0.13 | .040 |
