# Supplementary material for: Seven-Day Mortality Can Be Predicted in Medical Patients by Blood Pressure, Age, Respiratory Rate, Loss of Independence, and Peripheral Oxygen Saturation (the PARIS Score): A Prospective Cohort Study with External Validation
Source: PLoS One. 2015 Apr 13;10(4):e0122480. doi: 10.1371/journal.pone.0122480 (PMC4395094; doi:10.1371/journal.pone.0122480)
Supplement: S3 Table — (DOCX) [file pone.0122480.s004.docx]

**S3 Table - Performance measures using two alternative definitions of loss of independence, ie, ability to stand unaided and unable to get out of a chair unaided**

| Cohort | Discriminatory power | 95% confidence interval | Calibration |
| --- | --- | --- | --- |
| Unable to stand unaided | | | |
| Development | 0.87 | 0.82-0.92 | *P*=.9570 |
| First validation | 0.90 | 0.86-0.93 | *P*=.6498 |
| Unable to get out of chair unaided | | | |
| Development | 0.88 | 0.82-0.93 | *P*=.9498 |
| First validation | 0.90 | 0.87-0.93 | *P*=.6074 |
