# Supplementary material for: Seven-Day Mortality Can Be Predicted in Medical Patients by Blood Pressure, Age, Respiratory Rate, Loss of Independence, and Peripheral Oxygen Saturation (the PARIS Score): A Prospective Cohort Study with External Validation
Source: PLoS One. 2015 Apr 13;10(4):e0122480. doi: 10.1371/journal.pone.0122480 (PMC4395094; doi:10.1371/journal.pone.0122480)
Supplement: S4 Table — (DOCX) [file pone.0122480.s005.docx]

**S4 Table - Missing data in all three cohorts, data presented as number (%)**

| Variable | Development cohort | | First validation cohort | | Second validation cohort | |
| --- | --- | --- | --- | --- | --- | --- |
|  | Alive | Dead | Alive | Dead | Alive | Dead |
| Pulse | 7 (0.2) | 2 (2.6) | 43 (1.5) | 2 (3.5) | 60 (2.5) | 10 (9.0) |
| Systolic blood pressure | 6 (0.2) | 1 (1.3) | 38 (1.4) | 1 (1.8) | 64 (2.6) | 11 (9.9) |
| Temperature | 63 (2.1) | 6 (7.9) | 80 (2.9) | 2 (3.5) | 176 (7.2) | 10 (9.0) |
| SaO_2_/FiO_2_ | 83 (2.8) | 3 (4.0) | 98 (3.5) | 5 (8.8) | 211 (8.6) | 18 (16.2) |
| Respiratory rate | 896 (30.2) | 20 (26.3) | 338 (12.1) | 11 (19.3) | 413 (16.7) | 22 (19.8) |
| Blood glucose | 242 (8.2) | 8 (10.5) | 154 (5.5) | 4 (7.0) | 2450 (100.0) | 111 (100.0) |
| Level of consciousness | 15 (0.5) | 3 (4.0) | 40 (1.4) | 1 (1.8) | 56 (2.3) | 5 (4.5) |
| Loss of independence | 544 (18.3) | 13 (17.1) | 268 (9.6) | 5 (8.8) | 116 (4.7) | 5 (4.5) |
| Age | 0 (0.0) | 0 (0.0) | 0 (0.0) | 0 (0.0) | 0 (0.0) | 0 (0.0) |
