# Supplementary material for: Seven-Day Mortality Can Be Predicted in Medical Patients by Blood Pressure, Age, Respiratory Rate, Loss of Independence, and Peripheral Oxygen Saturation (the PARIS Score): A Prospective Cohort Study with External Validation
Source: PLoS One. 2015 Apr 13;10(4):e0122480. doi: 10.1371/journal.pone.0122480 (PMC4395094; doi:10.1371/journal.pone.0122480)
Supplement: S5 Table — (DOCX) [file pone.0122480.s006.docx]

**S5 Table - Logistic regression of the full model using list-wise deletion without multiple imputation**

| Variable | Coefficients | *P* value |
| --- | --- | --- |
| Systolic blood pressure (mmHg) | -0.024 (-0.037-0.010) | <.001 |
| Age (years) | 0.024 (0.0024-0.046) | .0029 |
| Respiratory rate (breaths/min) | 0.051 (0.012-0.090) | .0010 |
| SaO_2_/FiO_2_ (%/100) | -0.0042 (-0.0074-0.0009) | <.0001 |
| Loss of independence (yes/no) | 1.69 (0.92-2.47) | .0011 |
| Intercept | -2.65 (-5.42-0.12) | .0061 |
