# Supplementary material for: Seven-Day Mortality Can Be Predicted in Medical Patients by Blood Pressure, Age, Respiratory Rate, Loss of Independence, and Peripheral Oxygen Saturation (the PARIS Score): A Prospective Cohort Study with External Validation
Source: PLoS One. 2015 Apr 13;10(4):e0122480. doi: 10.1371/journal.pone.0122480 (PMC4395094; doi:10.1371/journal.pone.0122480)
Supplement: S6 Table — (DOCX) [file pone.0122480.s007.docx]

**S6 Table - Seven-day mortality in the simplified model in each of the three cohorts, number (%)**

| Score | Development cohort | | First validation cohort | | Second validation cohort | |
| --- | --- | --- | --- | --- | --- | --- |
|  | Alive | Dead | Alive | Dead | Alive | Dead |
| 0 | 724 (99.7) | 2 (0.3) | 1066 (99.9) | 1 (0.1) | 635 (99.8) | 1 (0.2) |
| 1 | 581 (99.3) | 4 (0.7) | 613 (99.0) | 6 (1.0) | 632 (98.9) | 7 (1.1) |
| 2 | 356 (98.1) | 7 (1.9) | 344 (97.7) | 2 (2.3) | 436 (95.4) | 21 (4.6) |
| 3 | 198 (90.8) | 20 (9.2) | 155 (91.7) | 14 (8.3) | 208 (90.0) | 23 (10.0) |
| 4 | 65 (84.4) | 12 (15.6) | 39 (78.0) | 11 (22.0) | 46 (65.7) | 24 (34.3) |
| 5 | 10 (66.7) | 5 (33.3) | 4 (100.0) | 0 (0.0) | 5 (38.5) | 8 (61.5) |
