# Supplementary material for: Seven-Day Mortality Can Be Predicted in Medical Patients by Blood Pressure, Age, Respiratory Rate, Loss of Independence, and Peripheral Oxygen Saturation (the PARIS Score): A Prospective Cohort Study with External Validation
Source: PLoS One. 2015 Apr 13;10(4):e0122480. doi: 10.1371/journal.pone.0122480 (PMC4395094; doi:10.1371/journal.pone.0122480)
Supplement: S7 Table — (DOCX) [file pone.0122480.s008.docx]

**S7 Table - Logistic regressions of the simplified score, both univariable and multivariable analyses; CI, confidence interval**

|  | Univariable analyses | |  | Multivariable analysis |  | |
| --- | --- | --- | --- | --- | --- | --- |
| Variable | Coefficient (95% CI) | Odds ratio (95% CI) | | Coefficient (95% CI) | | Odds ratio (95% CI) |
| Systolic blood pressure (≤115 mmHg) | 1.05 (0.48-1.62) | 2.87 (1.62-5.06) | | 0.73 (0.12-1.33) | | 2.07 (1.13-3.79) |
| Age (≥80 years) | 1.26 (0.70-1.83) | 3.54 (2.01-6.25) | | 0.52 (-0.089-1.14) | | 1.69 (0.91-3.12) |
| Respiratory rate (≥25 breaths/min) | 1.77 (1.20-2.34) | 5.87 (3.31-10.41) | | 0.88 (0.26-1.50) | | 2.42 (1.30-4.48) |
| SaO_2_ (≤93% or any supplementary oxygen) | 2.33 (1.53-3.14) | 10.31 (4.61-23.04) | | 1.38 (0.52-2.24) | | 3.97 (1.68-9.39) |
| Loss of independence (yes/no) | 2.61 (1.91-3.31) | 13.62 (6.76-27.46) | | 1.81 (1.06-2.56) | | 6.11 (2.88-13.0) |
| Intercept | - | - | | -6.13 (-7.01-5.25) | | - |
